# Supplementary material for: A comprehensive and quantitative exploration of thousands of viral genomes
Source: eLife. 2018 Apr 19;7:e31955. doi: 10.7554/eLife.31955 (PMC5908442; doi:10.7554/eLife.31955)
Supplement: Figure 3—source data 1. [file elife-31955-fig3-data1.docx]

|  |  | **Genome Length Statistics (kb)** | | | | | | |
| --- | --- | --- | --- | --- | --- | --- | --- | --- |
| **Classification** | **Classification Categories** | **Min** | **Max** | **25th Percentile** | **Median** | **75th Percentile** | **Mean** | **Stdev.** |
| **Host Domain** | Eukaryotic Viruses (N = 1384) | 1.3 | 2473.9 | 4.9 | 7.5 | 11.1 | 27.7 | 110.5 |
|  | Bacteria Viruses (N = 969) | 2.9 | 617.5 | 37.3 | 43.4 | 65.8 | 63.4 | 55.7 |
|  | Archaea Viruses (N = 46) | 7.0 | 95.7 | 15.7 | 24.4 | 40.9 | 31.7 | 21.9 |
| **Baltimore** | Group I (dsDNA) (N = 1211) | 4.8 | 2473.9 | 35.5 | 43.6 | 77.7 | 75.9 | 121.4 |
|  | Group II (ssDNA) (N = 431) | 1.3 | 11.7 | 2.7 | 2.8 | 5.3 | 4.0 | 1.8 |
|  | Group III (dsRNA) (N = 123) | 1.8 | 29.1 | 4.8 | 8.1 | 14.0 | 10.6 | 7.0 |
|  | Group IV (+ssRNA) (N = 482) | 2.6 | 33.5 | 6.6 | 8.5 | 10.1 | 9.6 | 5.5 |
|  | Group V (-ssRNA) (N = 101) | 8.9 | 19.2 | 11.1 | 11.9 | 15.4 | 13.0 | 2.6 |
|  | Group VI (ssRNA-RT) (N = 14) | 4.3 | 10.3 | 7.5 | 8.4 | 9.5 | 8.3 | 1.5 |
|  | Group VII (dsDNA-RT) (N = 37) | 3.0 | 8.8 | 7.3 | 7.5 | 7.8 | 6.8 | 1.8 |
| **Nucleotide Type** | DNA Viruses (N = 1679) | 1.3 | 2473.9 | 5.5 | 38.3 | 56.6 | 55.9 | 108.0 |
|  | RNA Viruses (N = 720) | 1.8 | 33.5 | 6.6 | 9.3 | 11.5 | 10.2 | 5.5 |
| **ICTV (orders)** | Caudovirales (N = 879) | 11.6 | 358.7 | 39.1 | 44.5 | 70.2 | 67.9 | 52.5 |
|  | Herpesvirales (N = 55) | 119.5 | 295.1 | 144.9 | 159.2 | 211.5 | 177.0 | 45.1 |
|  | Ligamenvirales (N = 11) | 20.9 | 41.2 | 31.8 | 36.9 | 40.4 | 34.7 | 6.5 |
|  | Mononegavirales (N = 71) | 8.9 | 19.2 | 11.4 | 12.0 | 15.5 | 13.4 | 2.5 |
|  | Nidovirales (N = 35) | 12.1 | 33.5 | 20.1 | 26.7 | 31.0 | 25.8 | 5.6 |
|  | Picornavirales (N = 89) | 6.6 | 14.3 | 7.8 | 8.4 | 9.8 | 8.9 | 1.4 |
|  | Tymovirales (N = 73) | 5.5 | 9.4 | 6.7 | 7.9 | 8.5 | 7.6 | 1.0 |
| **Combinations of different classifications** | All Eukaryotic dsDNA viruses  (N = 271) | 4.8 | 2473.9 | 7.3 | 33.0 | 152.7 | 109.0 | 232.5 |
|  | Baculoviridae (N = 22) | 84.3 | 176.7 | 108.6 | 127.6 | 151.1 | 127.2 | 23.9 |
|  | Poxviridae (N = 12) | 150.0 | 307.7 | 170.6 | 237.2 | 282.9 | 233.1 | 56.2 |
|  | Herpesvirales (N = 55) | 119.5 | 295.1 | 144.9 | 159.2 | 211.5 | 177.0 | 45.1 |
|  | Papillomaviridae (N = 73) | 7.0 | 8.3 | 7.3 | 7.6 | 7.7 | 7.6 | 0.3 |
|  | Adenoviridae (N = 31) | 26.3 | 45.8 | 31.6 | 35.1 | 43.4 | 36.1 | 6.0 |
|  | Polyomaviridae (N = 51) | 4.8 | 6.2 | 5.0 | 5.1 | 5.3 | 5.1 | 0.2 |
|  | All Bacterial dsDNA viruses  (N = 899) | 10.1 | 617.5 | 39.0 | 44.4 | 69.8 | 67.8 | 55.5 |
|  | Siphoviridae (N = 435) | 14.3 | 280.0 | 38.0 | 43.1 | 53.1 | 50.5 | 30.5 |
|  | Podoviridae (N = 200) | 11.7 | 145.9 | 39.2 | 42.5 | 50.4 | 47.2 | 19.5 |
|  | Myoviridae (N = 232) | 11.6 | 358.7 | 47.4 | 136.4 | 164.0 | 118.1 | 69.2 |
|  | All Archaeal dsDNA viruses (N = 41) | 8.1 | 95.7 | 17.4 | 28.3 | 41.2 | 34.5 | 21.6 |
|  | All Eukaryotic ssDNA viruses  (N = 375) | 1.3 | 8.1 | 2.7 | 2.8 | 5.2 | 3.5 | 1.4 |
|  | All Bacterial ssDNA viruses  (N = 51) | 4.4 | 11.7 | 5.8 | 6.8 | 7.8 | 6.8 | 1.5 |
