# Supplementary material for: A comprehensive and quantitative exploration of thousands of viral genomes
Source: eLife. 2018 Apr 19;7:e31955. doi: 10.7554/eLife.31955 (PMC5908442; doi:10.7554/eLife.31955)
Supplement: Figure 4—source data 1. — It is important to clarify that the median values in this table represent the median of median gene lengths. [file elife-31955-fig4-data1.docx]

|  |  | **Median Gene Length Statistics (bases)** | | | | | | |
| --- | --- | --- | --- | --- | --- | --- | --- | --- |
| **Classification** | **Classification Categories** | **Min** | **Max** | **25th Percentile** | **Median** | **75th Percentile** | **Mean** | **Stdev.** |
| **Host Domain** | Eukaryotic Viruses (N = 1384) | 272 | 22173 | 702 | 1055 | 2129 | 2192 | 2770 |
|  | Bacteria Viruses (N = 969) | 204 | 5100 | 366 | 408 | 456 | 429 | 192 |
|  | Archaea Viruses (N = 46) | 195 | 762 | 324 | 400 | 462 | 412 | 119 |
| **Baltimore** | Group I (dsDNA) (N = 1211) | 195 | 1577 | 380 | 429 | 555 | 539 | 269 |
|  | Group II (ssDNA) (N = 431) | 204 | 2777 | 404 | 588 | 774 | 692 | 419 |
|  | Group III (dsRNA) (N = 123) | 453 | 22173 | 1638 | 2291 | 3978 | 4148 | 4409 |
|  | Group IV (+ssRNA) (N = 482) | 297 | 17715 | 828 | 2366 | 6372 | 3742 | 3266 |
|  | Group V (-ssRNA) (N = 101) | 648 | 5052 | 1167 | 1353 | 1568 | 1448 | 633 |
|  | Group VI (ssRNA-RT) (N = 14) | 362 | 3530 | 1154 | 1799 | 2103 | 1805 | 921 |
|  | Group VII (dsDNA-RT) (N = 37) | 368 | 6537 | 477 | 558 | 915 | 873 | 998 |
| **Nucleotide Type** | DNA Viruses (N = 1679) | 195 | 6537 | 393 | 444 | 708 | 586 | 354 |
|  | RNA Viruses (N = 720) | 297 | 22173 | 1014 | 2072 | 4812 | 3452 | 3360 |
| **ICTV (orders)** | Caudovirales (N = 879) | 224 | 972 | 369 | 408 | 456 | 419 | 76 |
|  | Herpesvirales (N = 55) | 669 | 1382 | 978 | 1107 | 1200 | 1092 | 151 |
|  | Ligamenvirales (N = 11) | 315 | 462 | 342 | 372 | 429 | 384 | 45 |
|  | Mononegavirales (N = 71) | 648 | 1896 | 1055 | 1266 | 1367 | 1218 | 275 |
|  | Nidovirales (N = 35) | 297 | 4920 | 537 | 672 | 1056 | 1045 | 1007 |
|  | Picornavirales (N = 89) | 3375 | 10041 | 6372 | 7056 | 8232 | 6963 | 1580 |
|  | Tymovirales (N = 73) | 402 | 5103 | 554 | 693 | 1014 | 1183 | 1138 |
| **Combinations of different classifications** | All Eukaryotic dsDNA viruses (N = 271) | 272 | 1577 | 714 | 990 | 1179 | 958 | 271 |
|  | Baculoviridae (N = 22) | 582 | 843 | 647 | 672 | 711 | 680 | 57 |
|  | Poxviridae (N = 12) | 614 | 762 | 650 | 695 | 729 | 691 | 45 |
|  | Herpesvirales (N = 55) | 669 | 1382 | 978 | 1107 | 1200 | 1092 | 151 |
|  | Papillomaviridae (N = 73) | 272 | 1577 | 1170 | 1209 | 1338 | 1231 | 187 |
|  | Adenoviridae (N = 31) | 510 | 999 | 636 | 681 | 771 | 706 | 104 |
|  | Polyomaviridae (N = 51) | 639 | 1320 | 797 | 990 | 1055 | 930 | 158 |
|  | All Bacterial dsDNA viruses (N = 899) | 224 | 972 | 369 | 408 | 456 | 419 | 78 |
|  | Siphoviridae (N = 435) | 248 | 644 | 366 | 401 | 429 | 402 | 57 |
|  | Podoviridae (N = 200) | 231 | 972 | 378 | 426 | 480 | 438 | 100 |
|  | Myoviridae (N = 232) | 224 | 678 | 372 | 419 | 483 | 431 | 76 |
|  | All Archaeal dsDNA viruses (N = 41) | 195 | 762 | 315 | 396 | 459 | 405 | 120 |
|  | All Eukaryotic ssDNA viruses (N = 375) | 300 | 2777 | 404 | 732 | 806 | 741 | 426 |
|  | All Bacterial ssDNA viruses (N = 51) | 204 | 653 | 303 | 348 | 404 | 352 | 84 |
