# Supplementary material for: A comprehensive and quantitative exploration of thousands of viral genomes
Source: eLife. 2018 Apr 19;7:e31955. doi: 10.7554/eLife.31955 (PMC5908442; doi:10.7554/eLife.31955)
Supplement: Figure 5—source data 1. [file elife-31955-fig5-data1.docx]

|  |  | | **Percent Noncoding (DNA/RNA)** | | | | | | | |
| --- | --- | --- | --- | --- | --- | --- | --- | --- | --- | --- |
| **Classification** | | **Classification Categories** | **Min** | **Max** | **25th Percentile** | **Median** | **75th Percentile** | **Mean** | | **Stdev** |
| **Host Domain** | | Eukaryotic Viruses (N = 1384) | 0 | 93 | 5 | 10 | 15 | 11 | 9 | |
|  |  | Bacteria Viruses (N = 969) | 3 | 92 | 7 | 9 | 11 | 10 | 6 | |
|  |  | Archaea Viruses (N = 46) | 3 | 21 | 7 | 10 | 13 | 10 | 4 | |
| **Baltimore** | | Group I (dsDNA) (N = 1211) | 2 | 92 | 7 | 9 | 12 | 11 | 7 | |
|  |  | Group II (ssDNA) (N = 431) | 1 | 91 | 10 | 14 | 24 | 17 | 10 | |
|  |  | Group III (dsRNA) (N = 123) | 0 | 47 | 5 | 8 | 12 | 9 | 8 | |
|  |  | Group IV (+ssRNA) (N = 482) | 0 | 43 | 3 | 5 | 9 | 7 | 5 | |
|  |  | Group V (-ssRNA) (N = 101) | 2 | 20 | 4 | 7 | 10 | 8 | 4 | |
|  |  | Group VI (ssRNA-RT) (N = 14) | 7 | 93 | 15 | 16 | 19 | 24 | 23 | |
|  |  | Group VII (dsDNA-RT) (N = 37) | 0 | 31 | 9 | 11 | 14 | 11 | 6 | |
| **Nucleotide Type** | | DNA Viruses (N = 1679) | 0 | 92 | 8 | 10 | 14 | 12 | 8 | |
|  |  | RNA Viruses (N = 720) | 0 | 93 | 4 | 6 | 10 | 8 | 7 | |
| **ICTV (orders)** | | Caudovirales (N = 879) | 3 | 92 | 7 | 9 | 11 | 10 | 5 | |
|  |  | Herpesvirales (N = 55) | 2 | 38 | 16 | 19 | 22 | 19 | 6 | |
|  |  | Ligamenvirales (N = 11) | 8 | 21 | 9 | 12 | 17 | 13 | 4 | |
|  |  | Mononegavirales (N = 71) | 2 | 20 | 4 | 8 | 10 | 8 | 4 | |
|  |  | Nidovirales (N = 35) | 1 | 8 | 2 | 3 | 5 | 4 | 2 | |
|  |  | Picornavirales (N = 89) | 2 | 23 | 9 | 11 | 12 | 11 | 4 | |
|  |  | Tymovirales (N = 73) | 2 | 13 | 3 | 4 | 4 | 4 | 2 | |
| **Combinations of different classifications** | | All Eukaryotic dsDNA viruses (N = 271) | 2 | 86 | 8 | 11 | 16 | 14 | 9 | |
|  |  | Baculoviridae (N = 22) | 6 | 24 | 8 | 10 | 12 | 11 | 4 | |
|  |  | Poxviridae (N = 12) | 6 | 22 | 7 | 10 | 13 | 11 | 5 | |
|  |  | Herpesvirales (N = 55) | 2 | 38 | 16 | 19 | 22 | 19 | 6 | |
|  |  | Papillomaviridae (N = 73) | 5 | 51 | 8 | 9 | 12 | 11 | 8 | |
|  |  | Adenoviridae (N = 31) | 4 | 18 | 6 | 7 | 11 | 8 | 3 | |
|  |  | Polyomaviridae (N = 51) | 4 | 31 | 9 | 11 | 14 | 12 | 5 | |
|  |  | All Bacterial dsDNA viruses (N = 899) | 3 | 92 | 7 | 9 | 11 | 10 | 5 | |
|  |  | Siphoviridae (N = 435) | 3 | 39 | 7 | 9 | 11 | 10 | 5 | |
|  |  | Podoviridae (N = 200) | 3 | 55 | 7 | 9 | 10 | 10 | 6 | |
|  |  | Myoviridae (N = 232) | 3 | 92 | 7 | 9 | 11 | 10 | 6 | |
|  |  | All Archaeal dsDNA viruses (N = 41) | 3 | 21 | 7 | 10 | 13 | 10 | 4 | |
|  |  | All Eukaryotic ssDNA viruses (N = 375) | 1 | 80 | 10 | 14 | 24 | 17 | 10 | |
|  |  | All Bacterial ssDNA viruses (N = 51) | 4 | 91 | 8 | 14 | 19 | 16 | 14 | |
