# Supplementary material for: A comprehensive and quantitative exploration of thousands of viral genomes
Source: eLife. 2018 Apr 19;7:e31955. doi: 10.7554/eLife.31955 (PMC5908442; doi:10.7554/eLife.31955)
Supplement: Figure 8—source data 1. [file elife-31955-fig8-data1.docx]

| Accession Numbers | Genome names for pairs of viruses with similar attachment site sequences | Same host species? | Attachment site sequences |
| --- | --- | --- | --- |
| NC_028804 | Mycobacterium phage Barriga | yes | CTTGACACGTAACCGC |
| NC_028784 | Mycobacterium phage Tasp14 |  | CTTGACACGTAACCGC |
|  |  |  |  |
| NC_028804 | Mycobacterium phage Barriga | yes | CTTGACACGTAACCGC |
| NC_022329 | Mycobacterium phage PhrostyMug |  | CCTTGACACGTAACCGC |
|  |  |  |  |
| NC_028784 | Mycobacterium phage Tasp14 | yes | CTTGACACGTAACCGC |
| NC_022329 | Mycobacterium phage PhrostyMug |  | CCTTGACACGTAACCGC |
|  |  |  |  |
| NC_019921 | Staphylococcus phage phi5967PVL | yes | GGTCTTTTTTAAT |
| NC_012784 | Staphylococcus phage phiPVL-CN125 |  | TTTTTAATAAAA |
|  |  |  |  |
| NC_028764 | Clostridium phage phiCD505 | No, different phyla | GGTCTTTTTTAAT |
| NC_021789 | Cellulophaga phage phi19:3 |  | TTTTTAATAAAA |
|  |  |  |  |
| NC_007058 | Staphylococcus phage ROSA | No, but same Order | CGAACGTTTTGAAGA |
| NC_003291 | Listeria phage 2389 |  | GTTTTGAAGATAT |
|  |  |  |  |
| NC_013059 | Salmonella phage g341c | yes | TGGTATCAGGACGGAA |
| NC_011976 | Salmonella phage epsilon34 |  | TGGTATCAGGACGGAA |
|  |  |  |  |
| NC_027393 | Vibrio phage J2 | yes | GGGTGTGTCGAC |
| NC_005891 | Vibrio cholerae |  | GGGTGTGTCGAC |
|  |  |  |  |
| NC_027393 | Vibrio phage J2 | yes | GGGTGTGTCGAC |
| NC_005879 | Vibrio phage VP2 |  | GGGTGTGTCGAC |
|  |  |  |  |
| NC_005891 | Vibrio phage VP5 | yes | GGGTGTGTCGAC |
| NC_005879 | Vibrio phage VP2 |  | GGGTGTGTCGAC |
|  |  |  |  |
| NC_003315 | Haemophilus virus HP2 | yes | CTGGCGGTTATC |
| NC_001697 | Haemophilus virus HP1 |  | CTGGCGGTTATC |
|  |  |  |  |
| NC_022750 | Enterobacteria phage fiAA91-ss | No, but same Order | GGCGAGGCGGGGAAAGCAC |
| NC_004745 | Yersinia virus L413C |  | GGCGAGGCGGGGAAAGCAC |
|  |  |  |  |
| NC_022330 | Mycobacterium phage Quink | yes | GTTCGTCCCCGG |
| NC_022085 | Mycobacterium phage Goku |  | GTTCGTCCCCGG |
