# Supplementary material for: A comprehensive and quantitative exploration of thousands of viral genomes
Source: eLife. 2018 Apr 19;7:e31955. doi: 10.7554/eLife.31955 (PMC5908442; doi:10.7554/eLife.31955)
Supplement: Transparent reporting form [file elife-31955-transrepform.docx]

***eLife’s* transparent reporting form**

We encourage authors to provide detailed information *within their submission* to facilitate the interpretation and replication of experiments. Authors can upload supporting documentation to indicate the use of appropriate reporting guidelines for health-related research (see [EQUATOR Network](http://www.equator-network.org/%20)), life science research (see the [BioSharing Information Resource](https://biosharing.org/)), or the [ARRIVE guidelines](http://www.plosbiology.org/article/info:doi/10.1371/journal.pbio.1000412) for reporting work involving animal research. Where applicable, authors should refer to any relevant reporting standards documents in this form.

If you have any questions, please consult our Journal Policies and/or contact us: [editorial@elifesciences.org](mailto:editorial@elifesciences.org).

**Sample-size estimation**

- You should state whether an appropriate sample size was computed when the study was being designed
- You should state the statistical method of sample size computation and any required assumptions
- If no explicit power analysis was used, you should describe how you decided what sample (replicate) size (number) to use

Please outline where this information can be found within the submission (e.g., sections or figure legends), or explain why this information doesn’t apply to your submission:

We believe this information is not strictly relevant. The primary aim of this study is to describe the genomic properties of all viruses (N=2399) within the NCBI viral database (so long as the viral genome passes the inclusion criteria, see Methods).

**Replicates**

- You should report how often each experiment was performed
- You should include a definition of biological versus technical replication
- The data obtained should be provided and sufficient information should be provided to indicate the number of independent biological and/or technical replicates
- If you encountered any outliers, you should describe how these were handled
- Criteria for exclusion/inclusion of data should be clearly stated
- High-throughput sequence data should be uploaded before submission, with a private link for reviewers provided (these are available from both GEO and ArrayExpress)

Please outline where this information can be found within the submission (e.g., sections or figure legends), or explain why this information doesn’t apply to your submission:

- You should report how often each experiment was performed
  - There were no experiments performed.
- You should include a definition of biological versus technical replication
  - This does not apply because there were no biological or technical replicates.
- The data obtained should be provided and sufficient information should be provided to indicate the number of independent biological and/or technical replicates
  - See Data Availability in the Methods section. The Methods section details the logical steps for the code that was implemented while the annotated code underlying each figure is posted on GitHub (<https://github.com/gitamahm/VirologyByTheNumbers>).
- If you encountered any outliers, you should describe how these were handled
  - See Data Acquisition, Curation, and Statistical Analysis in the Methods section.
- Criteria for exclusion/inclusion of data should be clearly stated
  - The criteria for inclusion and exclusion of the data have been described in the introduction and in more detail within the Methods. The code shared on GitHub also lists the excluded genomes based on several listed criteria.
- High-throughput sequence data should be uploaded before submission, with a private link for reviewers provided (these are available from both GEO and ArrayExpress)
  - Any data derived from our analysis is provided in our code on GitHub, however our study did not generate high-throughput sequence data. We analyzed existing and publically-available, high-throughput sequence data from the NCBI viral database.

**Statistical reporting**

- Statistical analysis methods should be described and justified
- Raw data should be presented in figures whenever informative to do so (typically when N per group is less than 10)
- For each experiment, you should identify the statistical tests used, exact values of N, definitions of center, methods of multiple test correction, and dispersion and precision measures (e.g., mean, median, SD, SEM, confidence intervals; and, for the major substantive results, a measure of effect size (e.g., Pearson's r, Cohen's d)
- Report exact p-values wherever possible alongside the summary statistics and 95% confidence intervals. These should be reported for all key questions and not only when the p-value is less than 0.05.

Please outline where this information can be found within the submission (e.g., sections or figure legends), or explain why this information doesn’t apply to your submission:

- Statistical analysis methods should be described and justified
  - See Data Acquisition, Curation, and Statistical Analysis in the Methods section.
- Raw data should be presented in figures whenever informative to do so (typically when N per group is less than 10)
  - Our N values are typically much larger than 10, however, we show the raw data in all Figures, Tables and the SI (outliers are not removed).
- For each experiment, you should identify the statistical tests used, exact values of N, definitions of center, methods of multiple test correction, and dispersion and precision measures (e.g., mean, median, SD, SEM, confidence intervals; and, for the major substantive results, a measure of effect size (e.g., Pearson's r, Cohen's d)
  - See Methods and figure captions.
- Report exact p-values wherever possible alongside the summary statistics and 95% confidence intervals. These should be reported for all key questions and not only when the p-value is less than 0.05.
  - N values are reported in all figures and tables. All P values fall below 10^-5^ and have been denoted in figures and in text, whenever appropriate. Summary statistics are provided in Table 1 and in more detail in SI Tables 1-3.

(For large datasets, or papers with a very large number of statistical tests, you may upload a single table file with tests, Ns, etc., with reference to sections in the manuscript.)

**Group allocation**

- Indicate how samples were allocated into experimental groups (in the case of clinical studies, please specify allocation to treatment method); if randomization was used, please also state if restricted randomization was applied
- Indicate if masking was used during group allocation, data collection and/or data analysis

Please outline where this information can be found within the submission (e.g., sections or figure legends), or explain why this information doesn’t apply to your submission:

This information does not apply to our manuscript as we have not performed any experimentation.

**Additional data files (“source data”)**

- We encourage you to upload relevant additional data files, such as numerical data that are represented as a graph in a figure, or as a summary table
- Where provided, these should be in the most useful format, and they can be uploaded as “Source data” files linked to a main figure or table
- Include model definition files including the full list of parameters used
- Include code used for data analysis (e.g., R, MatLab)
- Avoid stating that data files are “available upon request”

Please indicate the figures or tables for which source data files have been provided:

Source data for all figures and tables are provided (<https://github.com/gitamahm/VirologyByTheNumbers>). Please refer to Data Availability in the Methods section for more detailed information.
